# Redox status regulates eggshell color by modulating protoporphyrin IX biosynthesis via the SIRT1/PGC-1α/ALAS1 axis in brown-shelled hens

**DOI:** 10.1186/s40104-025-01292-9

**Published:** 2025-11-21

**Authors:** Yu Fu, Mingyuan Lu, Dongkai Liu, Jianping Wang, Haijun Zhang, Guanghai Qi, Jing Wang

**Affiliations:** 1 Key Laboratory of Feed Biotechnology, Ministry of Agriculture and Rural Affairs, Institute of Feed Research, Chinese Academy of Agricultural Sciences, Beijing, 100081 China; 2 Key Laboratory of Animal Disease-Resistance Nutrition, Ministry of Education, Ministry of Agriculture and Rural Affairs, Key Laboratory of Sichuan Province, Animal Nutrition Institute, Sichuan Agricultural University, Chengdu, 611130 China

**Keywords:** Eggshell depigmentation, Mitochondrion, Oxidative stress, Protoporphyrin IX, SIRT1/PGC-1α

## Abstract

**Background:**

This study investigated the molecular mechanisms by which redox status regulates protoporphyrin IX (PpIX) biosynthesis and eggshell coloration in brown-shelled laying hens. This study consisted of two experiments involving 48 and 32 healthy 60-week-old Hy-Line Brown hens, respectively. The hens exhibited either dark (L* = 51.99 ± 2.08) or light (L* = 64.12 ± 3.02) brown eggshell colors. In Exp. 1, light brown-shelled hens were fed a basal diet (Lb group), while dark brown-shelled hens received either a basal diet (Db group) or a basal diet with 10 mg/kg ammonium metavanadate (Dbv group) for 20 d. In Exp. 2, light brown-shelled hens received either a basal diet (Lbc group) or a basal diet supplemented with 200 mg/kg resveratrol (Lbr group) for 12 weeks.

**Results:**

Compared to the Db group, eggshell L* values increased, and PpIX concentrations in both eggshell and uterus decreased in Dbv and Lb groups. These groups also showed oxidative stress, as indicated by reduced hepatic T-SOD and CAT activities. Uterine redox status changes were further confirmed by increased T-AOC level (Dbv) and reduced *CAT* gene expression (Lb). These redox disturbances led to reduced expression of *ND4* and *COX1* mtDNA, decreased ATP production and CS activity, along with upregulation of *IR*, *PI3K*, *HK*, and *PK* gene expression, reflecting altered mitochondrial energy metabolism. Notably, the SIRT1/PGC-1α signaling cascade and its downstream target ALAS1 were significantly downregulated at both mRNA and protein levels in Dbv and Lb groups. Compared to the Lbc group, the Lbr group exhibited higher antioxidant capacity by increasing hepatic CAT activity and uterine T-SOD and GSH-Px activities, and reducing MDA levels. Moreover, the Lbr group restored mitochondrial function and PpIX biosynthesis by upregulating *ND4* and *COX1* mtDNA, *CS* and *SDHA* gene expression, and SIRT1/PGC-1α/ALAS1 signaling, while downregulating LDH activity and the expression of *IR* and *PI3K*, thereby alleviating eggshell color fading.

**Conclusion:**

Oxidative stress induces eggshell depigmentation by impairing mitochondrial function and downregulating the SIRT1/PGC-1α/ALAS1 pathway, leading to reduced PpIX biosynthesis. Specifically, vanadium-induced or endogenous oxidative stress disrupts mitochondrial energy metabolism and suppresses key components of this pathway, while resveratrol alleviates oxidative damage and restores mitochondrial function and ALAS1-driven PpIX synthesis through reactivation of the SIRT1/PGC-1α axis.

**Supplementary Information:**

The online version contains supplementary material available at 10.1186/s40104-025-01292-9.

## Introduction

The eggshell color plays a crucial role in consumer purchasing intention, especially for brown eggs, as even slight fading can reduce their willingness to buy [1, 2]. The CIE L*a*b* color space system is typically used to assess the eggshell color, where the L* value represents brightness, with higher values indicating a lighter eggshell color [3]. In brown egg production, eggshell depigmentation typically exhibits an increased L* value, which is considered to be strongly associated with reduced levels of protoporphyrin IX (PpIX) [4]. PpIX is synthesized in the uterus, and its production and metabolism are influenced by multiple physiological and environmental factors. Among these, the redox status has emerged as a critical yet underexplored determinant [5, 6].

Mitochondria in uterine epithelial cells are the main sites of PpIX synthesis [7], and mitochondrial dysfunction has been identified as a major cause of reduced uterine PpIX levels [8]. Oxidative stress, a key driver of mitochondrial DNA damage and impaired energy metabolism [8, 9], may therefore critically affect PpIX synthesis in uterine epithelial cells, although the underlying mechanisms remain unclear.

A central regulator in the intersection of redox balance and mitochondrial function is Sirtuin 1 (SIRT1). Under oxidative stress, SIRT1 could restore the intracellular redox balance by regulating downstream proteins and pathways [10, 11]. Among these, a key target is peroxisome proliferator-activated receptor gamma coactivator 1-alpha (PGC-1α), a pivotal regulator of mitochondrial biogenesis and mitophagy [10, 12]. SIRT1 activates PGC-1α through deacetylation, thereby promoting mitochondrial turnover and enhancing mitochondrial function [13]. However, the activity of SIRT1 depends on the cellular NAD^+^ levels, which are often depleted during oxidative stress [14, 15], potentially leading to decreased SIRT1 expression and activity, ultimately compromising mitochondrial function [16, 17]. In addition to its role in mitochondrial regulation, PGC-1α is thought to influence the expression of aminolevulinic acid synthase 1 (ALAS1), the first and rate-limiting enzyme in the biosynthesis of PpIX. This regulation is proposed to occur through coactivation of nuclear respiratory factor 1 (Nrf1) [18, 19] and the insulin-sensitive transcription factor forkhead box protein O1 (FoxO1) [20]. Consequently, the inhibition of the SIRT1/PGC-1α axis caused by oxidative stress may suppress ALAS1 expression, leading to reduced PpIX synthesis.

Taken together, these evidences suggest that oxidative stress may suppress uterine pigment biosynthesis by disrupting mitochondrial homeostasis and downregulating the SIRT1/PGC-1α/ALAS1 signaling axis. To test this hypothesis, we employed both a vanadium-induced oxidative stress model and a naturally faded laying hen model (previously suggested to be potentially associated with oxidative stress [21]) to investigate their effects on antioxidant capacity, mitochondrial energy metabolism, and PpIX biosynthesis in the uterus. Furthermore, we evaluated whether resveratrol, a natural antioxidant and SIRT1 activator, could restore mitochondrial function and pigment synthesis in hens naturally laying light-colored eggs. These investigations aim to elucidate the mechanism by which redox status regulates eggshell pigmentation and explore potential nutritional strategies to mitigate pigment loss in brown eggs.

## Materials and methods

### Animals and experimental design

Animal procedures were approved by the management of the Animal Care and Use Committee of Institute of Feed Research, Chinese Academy of Agricultural Sciences. A total of 500 Hy-Line Brown laying hens at 60 weeks of age were caged individually. The environmental temperature was maintained at 22 °C with a light cycle of 16 h of light and 8 h of darkness. Hens were monitored for eggshell L* values daily over a 20-day period. Based on L* values deviating by two standard deviations from the mean, 32 hens producing dark brown eggs (average L* value = 51.99 ± 2.08) and 48 hens producing light brown eggs (average L* value = 64.12 ± 3.02) were selected for subsequent experiments.

#### Exp. 1

**Fig. 1:**
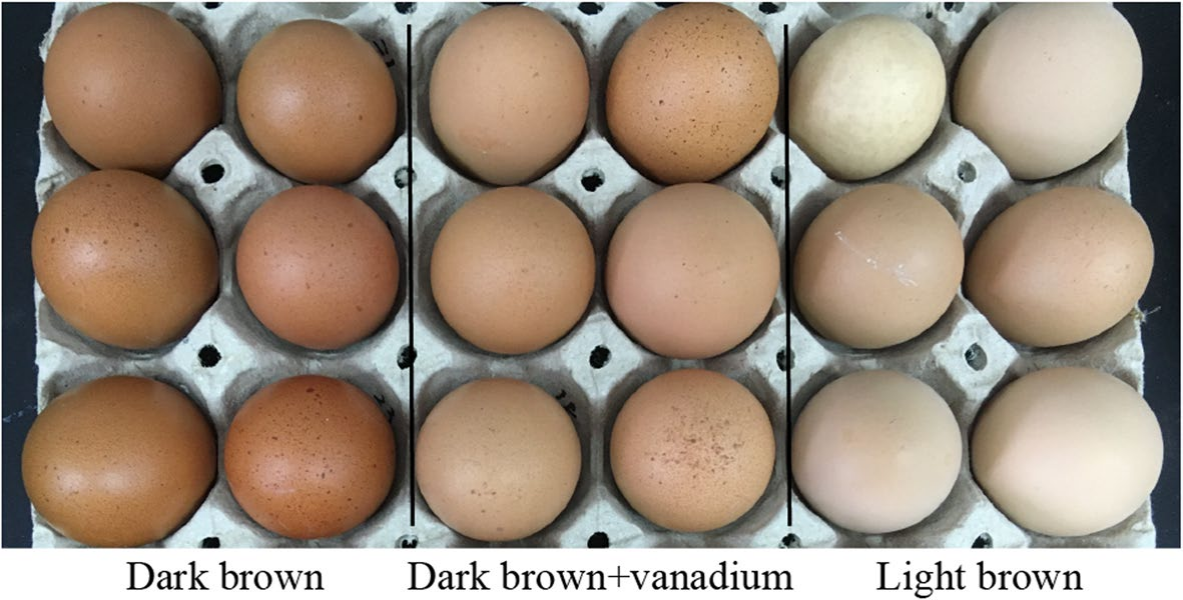
Representative photographs showing visible differences in eggshell color among the dark brown (Db), dark brown with vanadium supplementation (Dbv), and light brown (Lb) groups

Thirty-two hens producing dark brown eggs were randomly assigned to two groups: the dark brown (Db) group (*n* = 16), which received a basal diet, and the vanadium-treated (Dbv) group (*n* = 16), which was fed a basal diet supplemented with 10 mg/kg ammonium metavanadate. The addition of ammonium metavanadate was designed in reference to earlier studies, where its supplementation was shown to induce oxidative stress and reduce brown eggshell pigmentation in laying hens [5, 22]. In addition, 16 hens producing light brown eggs were fed a basal diet, serving as the light brown (Lb) group (*n* = 16). Each hen was treated as an individual replicate and experimental unit. The experimental period lasted 20 d to establish vanadium-induced oxidative stress and observe changes in eggshell color (Fig. 1). The basal diet (Table S1) met or slightly exceeded the nutritional requirements of the Chinese Feeding Standard of Chicken [23] and National Research Council guidelines [24].

#### Exp. 2

**Fig. 2:**
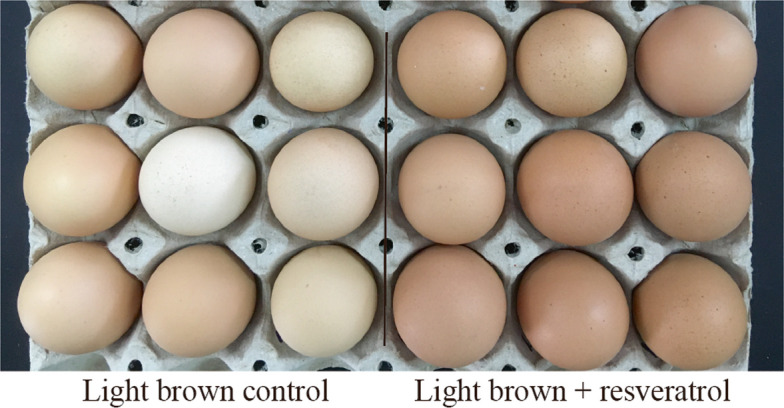
Representative photographs showing visible differences in eggshell color between the light brown control (Lbc) and light brown with resveratrol supplementation (Lbr) groups

Thirty-two hens producing light brown eggs were randomly assigned to two groups: resveratrol treatment (Lbr) group (*n* = 16), which was fed a basal diet supplemented with 200 mg/kg resveratrol, and the light brown control (Lbc) group (*n* = 16), which received a basal diet. The supplementation of resveratrol was designed in reference to earlier studies, which reported that resveratrol activates SIRT1 and enhances antioxidant capacity in laying hens [25, 26]. Each hen was then individually used as both a replicate and a test unit. The experimental period lasted 12 weeks to evaluate the sustained effects of resveratrol on eggshell pigmentation (Fig. 2). The basal diet was the same as that used in Exp. 1. Resveratrol (purity > 99.5%, A506244) was obtained from Sangon Biotech (Shanghai) Co., Ltd.

### Sample collection

In both Exp. 1 and Exp. 2, the last week was used as the observation period, during which the laying time of each hen was recorded daily. All hens were used for egg collection, while only hens with stable laying cycles were selected for tissue sampling. In Exp. 1, five eggs per hen (per replicate) collected during the last week were used for eggshell measurements. In Exp. 2, five eggs per hen (per replicate) were collected at weeks 1, 4, 8, and 12 for eggshell measurements. At the end of the trial, 8 birds per group were randomly selected and euthanized via cervical dislocation at 15 h post-oviposition. After euthanasia, only hens with an intact egg in the uterus undergoing shell pigmentation at this stage were included in the final sampling. Liver samples (left lobe, ~ 1 g) were harvested into cryopreservation tubes and stored at −80 °C for antioxidant index assays and RNA extraction. Uterus (eggshell gland) tissues (~ 1 g) were dissected, flash-frozen in liquid nitrogen, and stored at −80 °C for measurements of pigment concentration, antioxidant indices, RNA extraction, and total protein extraction.

### Eggshell quality

Eggshell color (L*, a*, and b* values), egg weight, eggshell breaking strength, and eggshell thickness were measured (16 replicates per group, 5 eggs per replicate). The egg weight was weighed first. Eggshell color was assessed at the equatorial region using a colorimeter (NH310, 3nh Co., Shenzhen, China), with the average of three measurements per egg was calculated. Eggshell breaking strength was determined with an egg force gauge (Israel Orka Food Technology Ltd., Ramat Hasharon, Israel). Eggshell thickness was measured at three locations on the egg surface (blunt end, equator, and sharp end) via an eggshell thickness gauge (Israel Orka Food Technology Ltd., Ramat Hasharon, Israel), and the mean value was used for analysis.

### Pigment concentrations in the uterine tissue and eggshells

The eggshell was cleaned and dried after the eggshell membrane was removed. The sample was ground into powder and passed through a 40-mesh sieve for the further detection. A vacuum freeze dryer (Shanghai Shenke Instrument Equipment Co., Shanghai, China) was used to lyophilize the uterine tissue, which was then ground into powder. The samples (0.25 g of eggshell powder or 0.1 g of freeze-dried uterine tissue powder) were placed in 10-mL centrifuge tubes. A total of 4 mL of pigment extraction solution (volume ratio of methanol to hydrochloric acid = 2:1, mixed evenly) was added, mixed and stored in the dark at 4 °C for 24 h. Then, the samples were centrifuged at 2,655 × *g* for 10 min, and 200 µL of the resulting supernatant was analyzed for PpIX and biliverdin contents via a microplate spectrophotometer (Molecular Devices Co., Shanghai, China) according to a previous report [8]. Each sample was analyzed in triplicate.

### RNA extraction and quantitative real-time PCR in the liver and uterine tissues

Approximately 80 mg of frozen uterus or liver was weighed and added to an RNase-free tube containing 1 mL of precooled TRNzol reagent (TIANGEN Biotech (Beijing) Co., Ltd., Beijing, China). All protocols were performed at 4 °C or on ice in accordance with the manufacturer’s instructions. Extracted RNA was dissolved in RNase-free water. The quantity and purity of total RNA were examined via a spectrophotometer (Amersham Bioscience, Sweden) and further assessed by gel electrophoresis. According to the manufacturer’s instructions, cDNA was synthesized from total RNA using the TIANGEN QuantScript RT kit (TIANGEN, Beijing, China). Quantitative real-time PCR was carried out using a SYBR Green PCR Master Mix kit (TIANGEN, Beijing, China) in CFX96 touch real-time PCR detection system (Bio-Rad Laboratories, CA, USA). The primer sequences for the target and reference genes are shown in Table S2. The protocol for real-time PCR reaction was as follows: 95 °C for 5 min, 40 cycles of 95 °C for 10 s, and 60 °C for 30 s. Each sample was analyzed in triplicate. The amplification efficiency of each gene was validated by constructing a standard curve through serial dilutions of cDNA. The specificity of the amplified products was confirmed by melting curves. β-Actin was used as the reference housekeeping gene for normalizing qPCR data. The relative mRNA expression of genes was calculated via the 2^−ΔΔCt^ method [27], as all primers showed similar amplification efficiencies close to 100%.

### Redox status in the liver and uterine tissues

Approximately 100 mg of tissue sample (liver or uterus) was weighed and placed into an EP tube. To this tube, 900 µL of 0.9% saline solution along with a steel bead was added. The tissue was then homogenized using a low-temperature tissue grinder for a duration of 2 min, followed by centrifugation at 12,850 × *g* for 10 min at 4 °C. The resulting supernatant was carefully collected for further analysis. The initial 10% liver tissue homogenate was subsequently diluted to a 5% solution using 0.9% saline. The activities of total antioxidant capacity (T-AOC), catalase (CAT), total superoxide dismutase (T-SOD), and glutathione peroxidase (GSH-Px), as well as the malondialdehyde (MDA) content, were measured via commercial assay kits (Nanjing Jiancheng Bioengineering Institute, Nanjing, China) according to the manufacturer’s protocols.

### Mitochondrial enzyme activity in the uterine tissue

After the same pretreatment procedure described in the “Redox status in the liver and uterine tissues”, the activities of citrate synthase (CS), lactate dehydrogenase (LDH), and succinate dehydrogenase (SDH) in the uterine samples were measured via assay kits purchased from Nanjing Jiancheng Bioengineering Institute following the manufacturer’s instructions.

### Relative mitochondrial DNA copy number in the uterine tissue

Mitochondrial DNA (mtDNA) was extracted from approximately 30 mg of frozen uterine tissue via the TIANamp Genomic DNA Kit (TIANGEN, Beijing, China) according to the manufacturer's instructions. The relative mtDNA expression was quantified via quantitative real-time PCR with a SYBR green kit on a CFX96 touch real-time PCR detection system (Bio-Rad Laboratories, CA, USA). The relative expression was calculated via the 2^−ΔΔCt^ method [27]. β-Actin was used as the reference housekeeping gene for normalizing qPCR data. The primer sequences for the target and reference genes are provided in Table S2.

### ATP content in the uterine tissue

Approximately 50 mg of thawed uterine samples were finely minced and transferred to an EP tube containing 450 µL of boiling double-distilled water. The mixture was vortexed in boiling water for 10 min, followed by centrifugation at 2,655 × *g* for 10 min. The resulting supernatant was collected and diluted to a final concentration of 1%. The ATP content was then quantified using a commercial ATP assay kit (Nanjing Jiancheng Bioengineering Institute, Nanjing, China) following the manufacturer’s protocol.

### Western blot analysis in the uterine tissue

Western blot was performed as described in our previous report [28]. Briefly, total proteins were extracted with lysis buffer containing Tris, NaCl, Triton X-100, and protease/phosphatase inhibitors. Then, protein concentrations were determined using a BCA assay kit (Beyotime, Shanghai, China), and 20 µg of protein per sample was loaded per lane. Proteins were separated by gel electrophoresis at a constant power of 6 W for 60 min and transferred to a PVDF membrane (Bio-Rad, CA, USA). The membrane was blocked with 5% non-fat dry milk in TBS-T (0.1% Tween-20) for 45 min at room temperature with agitation, followed by overnight incubation at 4 °C with primary antibodies: SIRT1 (Abcam, ab189494), ALAS1 (Abcam, ab154860), PGC1α (Abcam, ab54481), and GAPDH (Abcam, ab181602, used as a loading control). Further details of the antibodies, including host species, working dilution, and sequence homology, are provided in Table S3. After washing, the membrane was incubated with appropriate secondary antibodies at room temperature, then washed three times with TBST solution. The bands were developed using ECL substrate for 5 min and visualized with a Bio-Rad imaging system. Band intensities were quantified using ImageJ.

### Statistical analysis

All data were analyzed using SAS 9.4 (SAS Institute, Cary, NC, USA). Normality (Shapiro-Wilk test) and homogeneity of variance were assessed prior to analysis, with log2 transformation applied when assumptions were not met. Each hen was taken as an analysis unit. Sample size (*n* = 16 hens/group) was based on effect-size estimates from a previous study and provided > 80% power to detect the prespecified difference [29]. In Exp. 1, data were analyzed using one-way ANOVA, followed by Tukey’s HSD test for the comparisons among groups. In Exp. 2, eggshell traits measured at four time points (weeks 1, 4, 8, and 12) were analyzed by repeated-measures ANOVA with group and time as fixed factors and hen as a random effect; other traits were analyzed by *t*-test. Differences were considered statistically significant at a *P*-value < 0.05. The data are presented as the means ± SD (standard deviation).

## Results

### Laying performance and eggshell quality

The effects of vanadium on laying performance are listed in Table S4. The average daily feed intake and laying performance ranged from 94.50 to 105.50 g and 94.38% to 95.31%, respectively, with no significant differences observed among the groups. The effects of vanadium on eggshell quality are presented in Table 1. The eggshell L* values were significantly higher in the Dbv and Lb groups than in the Db group (*P* < 0.05), while the Dbv group exhibited a significantly lower L* value compared to the Lb group (*P* < 0.05). In addition, both the Dbv and Db groups showed higher eggshell a* and b* values compared with the Lb group, and the a* value was further elevated in the Db group relative to the Dbv group (*P* < 0.05). The differences were also visually discernible, as illustrated by representative photographs provided in Fig. 1. However, there were no significant differences in the egg weight, eggshell breaking strength, and eggshell thickness among the Db, Dbv, and Lb groups (*P* > 0.05).

**Table 1:** Effects of dietary vanadium addition on eggshell quality in Hy-line Brown laying hens^1^

Items	Db	Dbv	Lb	SEM	*P*-value
Egg weight, g	60.21	57.17	60.50	0.64	0.06
Eggshell breaking strength, N	34.69	34.64	37.16	0.69	0.23
Eggshell thickness, ×10^−2^ mm	44.17	45.57	44.65	0.51	0.52
L* value	51.24^c^	57.14^b^	64.12^a^	0.90	< 0.01
a* value	20.87^a^	18.76^b^	14.38^c^	0.44	< 0.01
b* value	25.47^a^	24.61^a^	22.61^b^	0.28	< 0.01

^1^Data represent the mean of 16 replicates, each with 5 eggs. *Db* Dark brown group, *Dbv* Vanadium-treated group, *Lb* Light brown group, *SEM* Standard error of the mean

^a–c^Values with no common letters differ significantly (*P* < 0.05)

The effects of resveratrol on laying performance are presented in Table S5. No significant differences were observed in the average daily feed intake and laying performance between the Lbc and Lbr groups (*P* > 0.05). The effects of dietary supplementation with resveratrol on eggshell quality are shown in Table 2. There were no significant differences on egg weight, eggshell breaking strength, eggshell thickness and eggshell b* value between the Lbc and Lbr groups (*P* > 0.05). However, the L* values were decreased at weeks 8 and 12, and a* values were increased at weeks 4, 8, and 12 in the Lbr group (*P* < 0.05). Representative photographs are shown in Fig. 2.

**Table 2:** Effects of dietary resveratrol addition on eggshell quality in Hy-line Brown laying hens^1^

Items^1^	Time, weeks	Lbc	Lbr	*P*-value		
				Group	Time	Group × Time
Egg weight, g	1	60.39 ± 2.14	60.23 ± 3.20			
	4	60.74 ± 2.72	60.70 ± 3.91			
	8	61.24 ± 2.72	60.45 ± 3.91			
	12	61.80 ± 2.64	61.94 ± 3.00	0.83	< 0.001	0.45
Eggshell breaking strength, N	1	35.66 ± 5.51	35.68 ± 7.55			
	4	35.19 ± 4.12	33.56 ± 6.08			
	8	35.73 ± 4.78	34.33 ± 6.33			
	12	35.10 ± 5.52	37.10 ± 5.43	0.89	0.25	0.19
Eggshell thickness, ×10^−2^ mm	1	44.83 ± 3.15	46.73 ± 2.20			
	4	42.93 ± 3.79	42.82 ± 3.65			
	8	43.73 ± 4.27	45.11 ± 2.15			
	12	44.07 ± 2.51	46.60 ± 2.87	0.11	< 0.001	0.16
L* value	1	64.27 ± 3.12	64.30 ± 2.31			
	4	64.69 ± 3.11	63.07 ± 2.70			
	8	65.75 ± 3.05^a^	63.03 ± 2.45^b^			
	12	65.49 ± 3.45^a^	62.05 ± 2.70^b^	0.046	0.37	< 0.001
a* value	1	15.65 ± 1.68	16.36 ± 1.22			
	4	14.46 ± 1.52^b^	15.60 ± 1.36^a^			
	8	13.52 ± 1.46^b^	16.02 ± 1.33^a^			
	12	14.49 ± 1.69^b^	15.81 ± 1.35^a^	0.005	< 0.001	< 0.001
b* value	1	23.84 ± 1.80	24.25 ± 1.29			
	4	22.82 ± 1.96	22.92 ± 1.46			
	8	22.61 ± 1.84	23.56 ± 1.25			
	12	23.01 ± 1.79	23.32 ± 2.00	0.43	< 0.001	0.19

^1^Data are presented as the mean ± standard deviation (SD) of 16 replicates, each with 5 eggs. *Lbc* Light brown control group, *Lbr* Light brown with resveratrol treatment group

^a,b^Values with no common letters differ significantly (*P* < 0.05)

### Pigment concentrations in eggshells and the uterus

The effects of vanadium on the pigment concentrations of eggshells and uterus are shown in Table 3. PpIX and biliverdin are major eggshell pigments, with PpIX appearing to play a more important role in brown eggshell coloration. The levels of PpIX in the eggshell and uterus, along with the uterine biliverdin concentration, were significantly lower in the Dbv and Lb groups than in the Db group (*P* < 0.05). Moreover, we observed significantly lower eggshell PpIX and uterine biliverdin levels in the Lb group compared with the Dbv group (*P* < 0.05).

**Table 3:** Effects of dietary vanadium addition on pigment concentrations in eggshells and the uterus of Hy-line Brown laying hens^1^

Samples	Items	Db	Dbv	Lb	SEM	*P*-value
Eggshell	Protoporphyrin IX, μg/g	140.11^a^	103.93^b^	53.12^c^	5.64	< 0.01
	Biliverdin, μg/g	8.46	7.93	7.81	0.29	0.63
Uterus	Protoporphyrin IX, μg/g	197.86^a^	163.50^b^	139.20^b^	7.34	0.01
	Biliverdin, μg/g	172.99^a^	81.56^b^	67.63^c^	11.06	< 0.01

^1^Data represent the mean of 16 replicates for the eggshell or 8 replicates for the uterus. *Db* Dark brown group, *Dbv* Vanadium-treated group, *Lb* Light brown group, *SEM* Standard error of the mean

^a–c^Values with no common letters differ significantly (*P* < 0.05)

The effects of dietary resveratrol supplementation on the pigments’ concentration of eggshells and the uterus are demonstrated in Table 4. The concentrations of eggshell biliverdin did not differ significantly between the Lbc and Lbr groups (*P* > 0.05). However, compared to the Lbc group, the eggshell PpIX concentration in the Lbr group was significantly increased at weeks 4, 8, and 12 (*P* < 0.05). By the end of the trial, the levels of uterine PpIX and biliverdin were significantly higher in the Lbr group than in the Lbc group (*P* < 0.05).

**Table 4:** Effects of dietary resveratrol addition on pigment concentrations in eggshells and the uterus of Hy-line Brown laying hens^1^

Items^1^	Time, weeks	Lbc	Lbr	*P*-value		
				Group	Time	Group × Time
Protoporphyrin IX, μg/g	1	61.90 ± 11.49	61.62 ± 11.30			
	4	56.99 ± 12.42^b^	71.58 ± 10.81^a^			
	8	53.12 ± 14.43^b^	72.69 ± 12.43^a^			
	12	52.17 ± 11.42^b^	76.26 ± 12.06^a^	< 0.001	0.82	0.001
Biliverdin, μg/g	1	7.75 ± 1.76	7.66 ± 2.25			
	4	8.70 ± 1.64	8.58 ± 1.25			
	8	7.81 ± 1.99	8.49 ± 0.83			
	12	7.81 ± 1.99	8.78 ± 1.48	0.98	0.11	0.81
Protoporphyrin IX, μg/g	12	139.20 ± 21.11^b^	162.73 ± 18.56^a^	0.033	-	-
Biliverdin, μg/g	12	57.64 ± 9.81^b^	75.15 ± 7.67^a^	0.001	-	-

^1^Data are presented as the mean ± standard deviation (SD) of 16 replicates for the eggshells or 8 replicates for the uterus. *Lbc* Light brown control group, *Lbr* Light brown with resveratrol treatment group

^a,b^Values with no common letters differ significantly (*P* < 0.05)

### Antioxidant enzyme activities and mRNA expressions in the liver

**Fig. 3:**
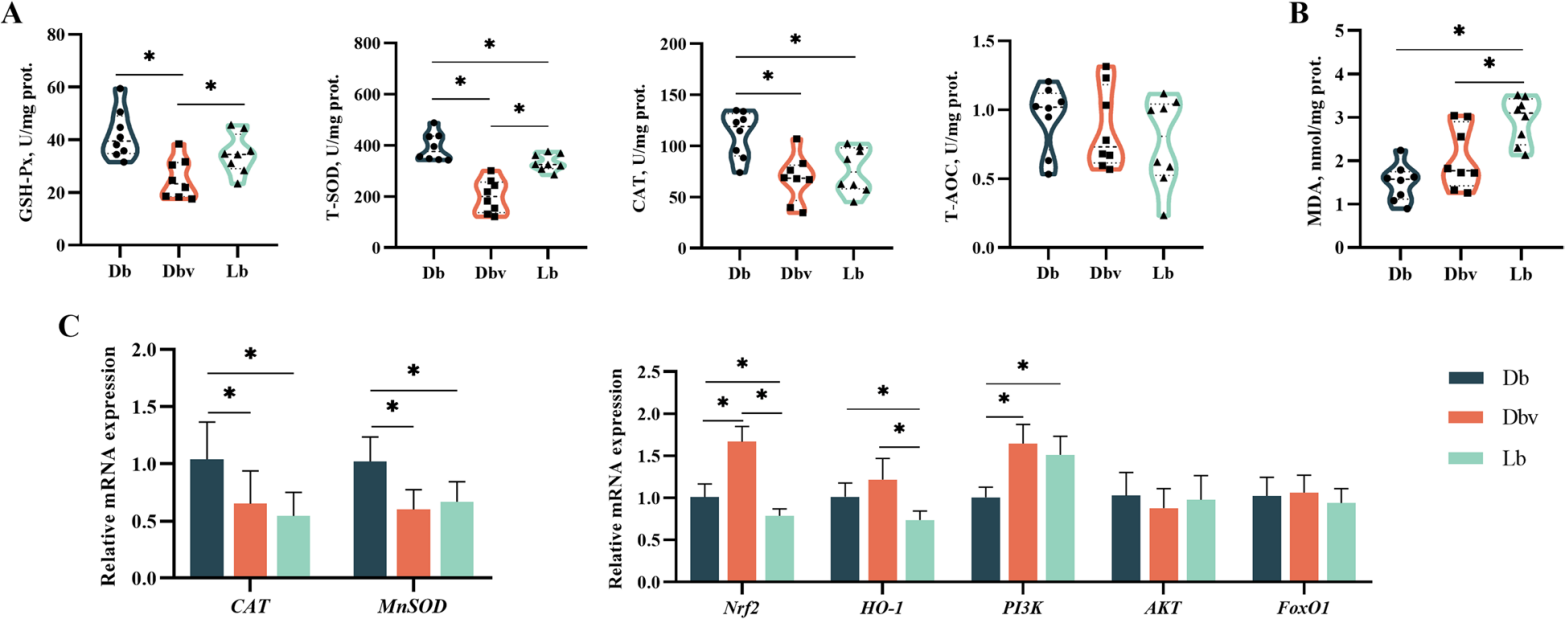
Effects of dietary vanadium addition on the antioxidant status of the liver in Hy-line Brown laying hens (*n* = 8). **A** The antioxidant enzyme (GSH-Px, T-SOD, CAT, and T-AOC) activities. **B** The MDA level. **C** The relative expression levels of antioxidant genes. GSH-Px, glutathione peroxidase; T-SOD, total superoxide dismutase; CAT, catalase; T-AOC, total antioxidant capacity; MDA, malondiadehyde; *MnSOD*, manganese superoxide dismutase; *Nrf2*, nuclear factor E2-related factor 2; *HO-1*, heme oxygenase 1; *PI3K*, phosphatidylinositol 3-kinase; *AKT*, serine/threonine kinase 1; *FoxO1*, forkhead box protein O1; Db, the dark brown group; Dbv, the vanadium-treated group; Lb, the light brown group. Data represent means with standard deviation based on 8 replicates. Asterisk (*) indicates significant difference (*P* < 0.05)

The effects of vanadium on the activities and levels of antioxidant enzymes in the liver are depicted in Fig. 3. Compared to Db and Lb groups, hepatic GSH-Px activity was significantly reduced in the Dbv group (*P* < 0.05). The Lb group exhibited higher hepatic T-SOD activity than the Dbv group, although both were lower than that of the Db group (*P* < 0.05). Hepatic CAT activity was significantly decreased in both Dbv and Lb groups compared to the Db group (*P* < 0.05). Compared to the Db and Dbv groups, the liver MDA level was significantly increased in the Lb group (*P* < 0.05). Compared to the Db group, the Dbv and Lb groups showed significantly downregulated expression levels of *CAT* and *MnSOD* genes, while the expression of the *PI3K* gene was significantly upregulated (*P* < 0.05). The expression of *Nrf2* gene was significantly increased in the Dbv group compared to the Db and Lb groups (*P* < 0.05), whereas it was significantly reduced in the Lb group relative to the Db group (*P* < 0.05). The expression of *HO-1* gene was significantly downregulated in the Lb group compared with the Db and Dbv groups (*P* < 0.05). However, no significant differences were observed in T-AOC activity or in the expression levels of *AKT* and *FoxO1* genes among the three groups (*P* > 0.05).

**Fig. 4:**
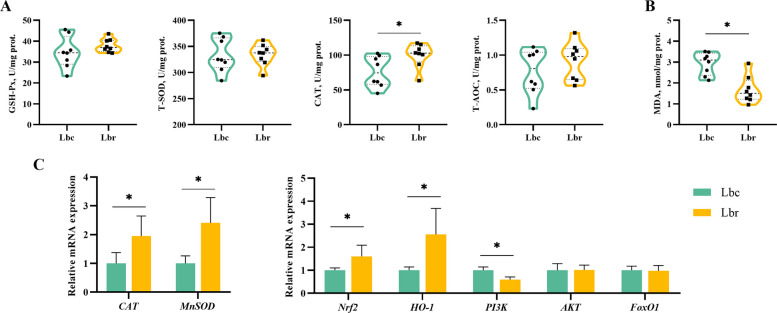
Effects of dietary resveratrol supplementation on liver antioxidant status in light brown-shelled laying hens (*n* = 8). **A** The antioxidant enzyme (GSH-Px, T-SOD, CAT, and T-AOC) activities. **B** The MDA level. **C** The relative expression levels of antioxidant genes. GSH-Px, glutathione peroxidase; T-SOD, total superoxide dismutase; CAT, catalase; T-AOC, total antioxidant capacity; MDA, malondiadehyde; *MnSOD*, manganese superoxide dismutase; *Nrf2*, nuclear factor E2-related factor 2; *HO-1*, heme oxygenase 1; *PI3K*, phosphatidylinositol 3-kinase; *AKT*, serine/threonine kinase 1; *FoxO1*, forkhead box protein O1; Lbc, the light brown control group; Lbr, the light brown with resveratrol treatment group. Data represent means with standard deviation based on 8 replicates. Asterisk (*) indicates a significant difference (*P* < 0.05)

Figure 4 illustrates the effects of dietary resveratrol supplementation on the antioxidant capacity of the liver. Compared to the Lbc group, the Lbr group had higher CAT activity and lower MDA level in the liver (*P* < 0.05). Dietary supplementation with resveratrol upregulated the expression of *CAT*, *MnSOD*, *Nrf2*, and *HO-1* genes, and downregulated the expression of the *PI3K* gene (*P* < 0.05). However, resveratrol supplementation did not significantly affect the activities of GSH-Px, T-SOD, and T-AOC, nor the expression levels of *AKT* and *FoxO1* genes (*P* > 0.05).

### Antioxidant enzyme activities and mRNA expressions in the uterus

**Fig. 5:**
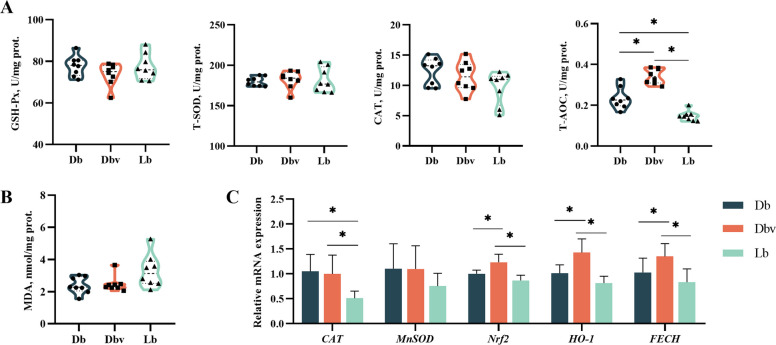
Effects of vanadium addition on the uterine antioxidant status in Hy-line Brown laying hens (*n* = 8). **A** The antioxidant enzyme (GSH-Px, T-SOD, CAT, and T-AOC) activities. **B** The MDA level. **C** The relative expression levels of antioxidant genes. GSH-Px, glutathione peroxidase; T-SOD, total superoxide dismutase; CAT, catalase; T-AOC, total antioxidant capacity; MDA, malondiadehyde; *MnSOD*, manganese superoxide dismutase; *Nrf2*, nuclear factor E2-related factor 2; *HO-1*, heme oxygenase 1; *FECH*, ferrochelatase; Db, the dark brown group; Dbv, the vanadium-treated group; Lb, the light brown group. Data represent means with standard deviation based on 8 replicates. Asterisk (*) indicates a significant difference (*P* < 0.05)

Figure 5 demonstrates the effects of vanadium addition on uterine antioxidant capacity. Compared to the Db group, T-AOC activity was increased in the Dbv group but decreased in the Lb group (*P* < 0.05). However, no significant differences were observed in the uterine GSH-Px, T-SOD, and CAT activities among three groups (*P* > 0.05). Compared to the Db group, the Dbv group significantly upregulated the expression of *Nrf2*, *HO-1*, and *FECH* genes, while the Lb group showed decreased expression of *CAT* gene (*P* < 0.05). The Dbv group exhibited higher expression of *CAT*, *Nrf2*, *HO-1*, and *FECH* genes compared to the Lb group (*P* < 0.05).

**Fig. 6:**
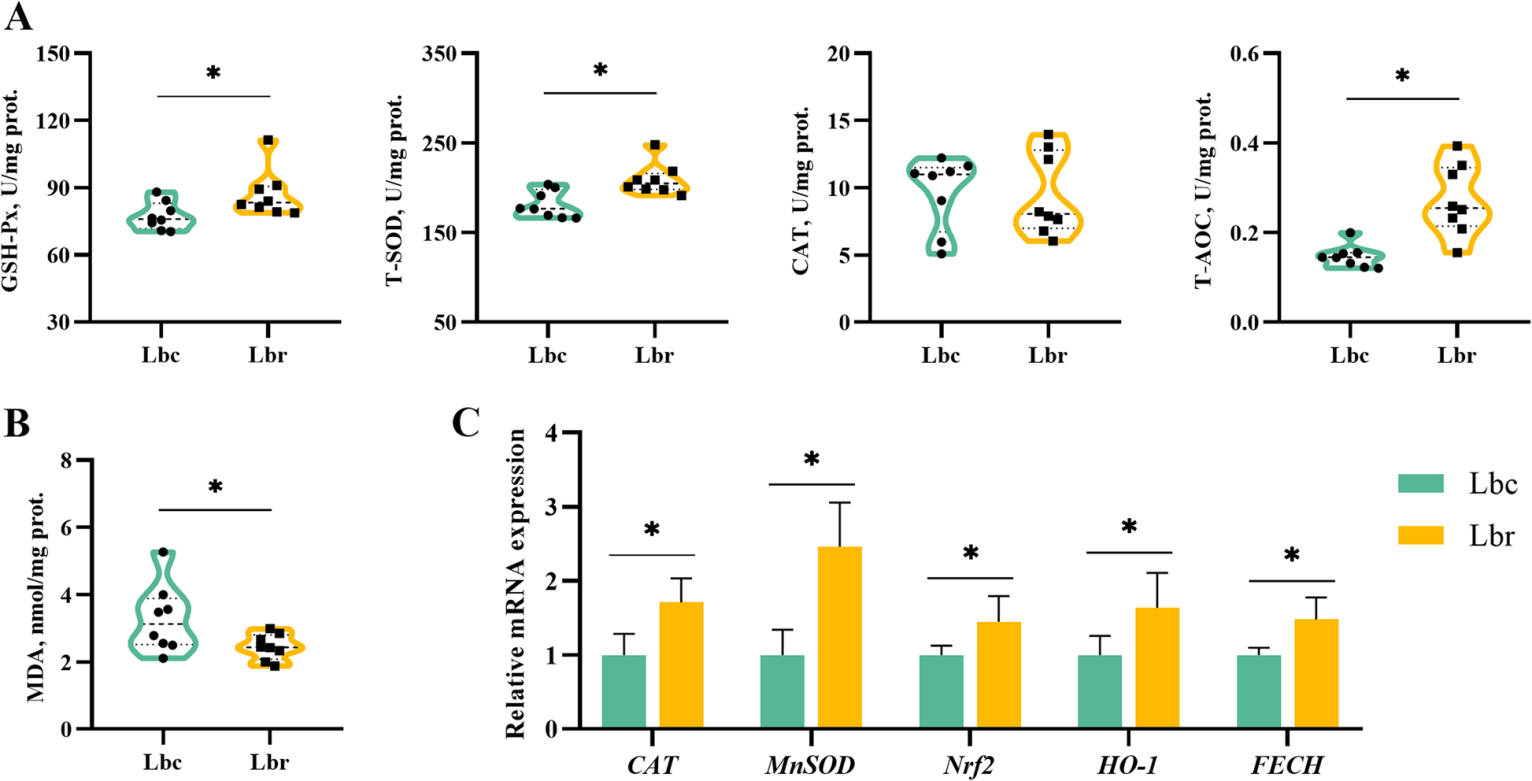
Effects of dietary resveratrol supplementation on the uterine antioxidant status in the light brown-shelled laying hens (*n* = 8). **A** The antioxidant enzyme (GSH-Px, T-SOD, CAT, and T-AOC) activities. **B** The MDA level. **C** The relative expression levels of antioxidant genes. GSH-Px, glutathione peroxidase; T-SOD, total superoxide dismutase; CAT, catalase; T-AOC, total antioxidant capacity; MDA, malondiadehyde; *MnSOD*, manganese superoxide dismutase; *Nrf2*, nuclear factor E2-related factor 2; *HO-1*, heme oxygenase 1; *FECH*, ferrochelatase; Lbc, the light brown control group; Lbr, the light brown with resveratrol treatment group. Data represent means with standard deviation based on 8 replicates. Asterisk (*) indicates a significant difference (*P* < 0.05)

The influences of dietary resveratrol supplementation on the antioxidant capacity of the uterus are shown in the Fig. 6. Compared to the Lbc group, resveratrol addition increased the activities of GSH-Px, T-SOD, and T-AOC in the uterus (*P* < 0.05). The MDA level was lower in the Lbr group than in the Lbc group (*P* < 0.05). Additionally, resveratrol supplementation upregulated the expression of *CAT*, *MnSOD*, *Nrf2*, *HO-1*, and *FECH* genes (*P* < 0.05).

### Mitochondrial function-related enzymes and genes

**Fig. 7:**
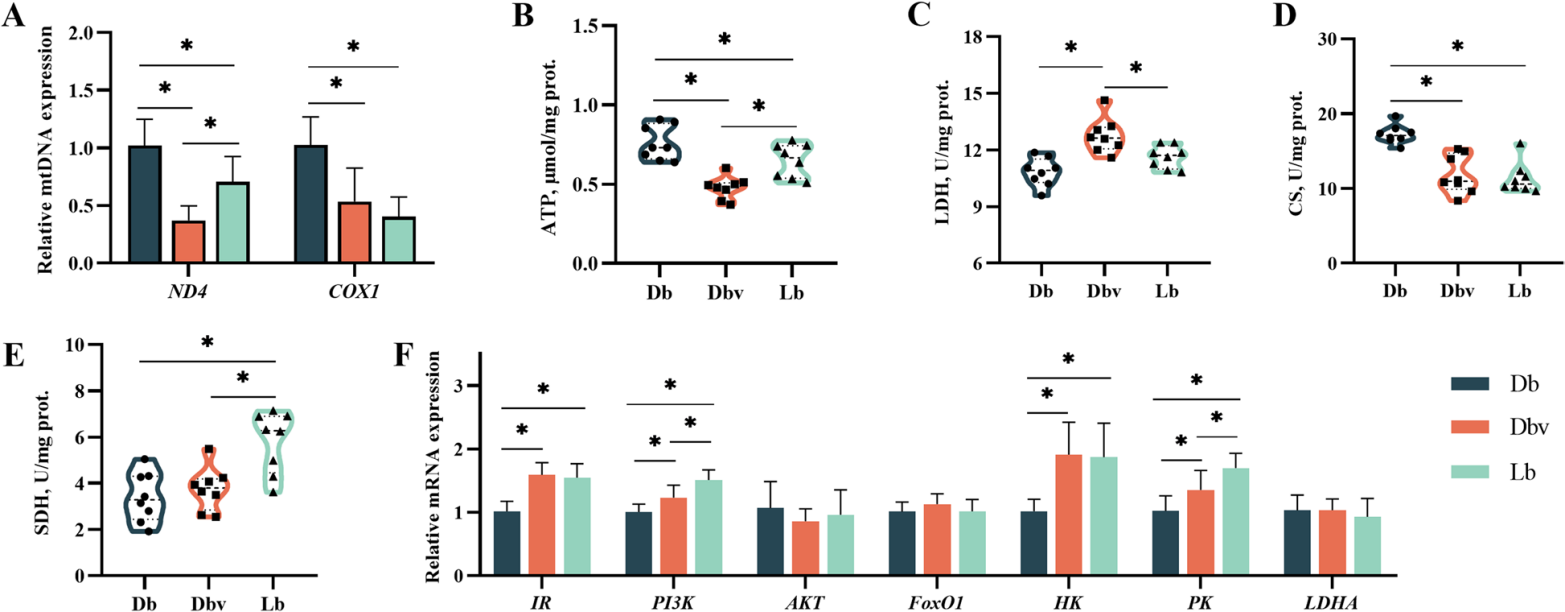
Effects of vanadium addition on mitochondrial function in the uterus (*n* = 8). **A** The relative expression of mitochondrial DNA (mtDNA). **B** ATP level. **C** LDH activity. **D** CS activity. **E** SDH activity. **F** The relative mRNA expression of genes related to mitochondrial function. *ND4*, nicotinamide adenine dinucleotide dehydrogenase subunit 4; *COX1*, cytochrome c oxidase 1; ATP, adenosine triphosphate; LDH, lactate dehydrogenase; CS, citrate synthase; SDH, succinate dehydrogenase; *IR*, insulin receptor; *PI3K*, phosphatidylinositol 3-kinase; *AKT*, serine/threonine kinase 1; *FoxO1*, forkhead box protein O1; *HK*, hexokinase; *PK*, pyruvate kinase; *LDHA*, lactate dehydrogenase A; Db, the dark brown group; Dbv, the vanadium-treated group; Lb, the light brown group. Asterisk (*) indicates a significant difference (*P* < 0.05)

The effects of vanadium addition on uterine mitochondrial function are shown in Fig. 7. Compared to the Db group, the expression levels of mtDNA *ND4* and *COX1* were downregulated in both Dbv and Lb groups (*P* < 0.05). Vanadium addition decreased the expression of mtDNA *ND4* compared to the Lb group (*P* < 0.05). Additionally, compared to the Db group, the Dbv group exhibited reduced ATP levels and CS activity, but increased LDH activity. Similarly, compared to the Lb group, the Dbv group showed lower ATP level and SDH activity, along with higher LDH activity (*P* < 0.05). Furthermore, the Lb group had lower ATP levels and CS activity, but higher SDH activity than the Db group (*P* < 0.05). Compared to the Db group, the expression levels of *IR*, *PI3K*, *HK*, and *PK* genes were elevated in both the Dbv and Lb groups, with the Lb group showing higher *PI3K* and *PK* expression than the Dbv group (*P* < 0.05). However, the expression levels of *AKT*, *FoxO1*, and *LDHA* genes were comparable among the three groups (*P* > 0.05).

**Fig. 8:**
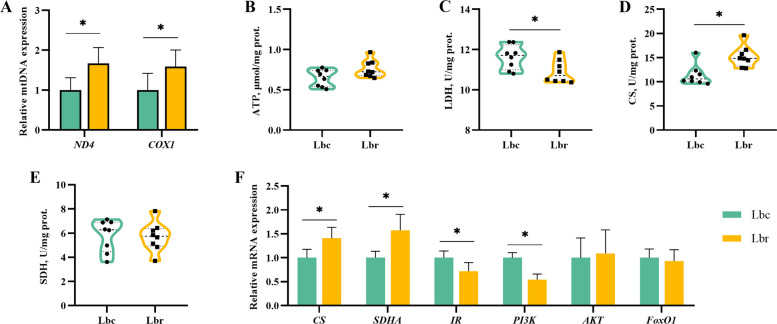
Effects of dietary resveratrol supplementation on mitochondrial function in the uterus (*n* = 8). **A** The relative expression of mitochondrial DNA (mtDNA). **B** ATP level. **C** LDH activity. **D** CS activity. **E** SDH activity. **F** The relative mRNA expression of genes related to mitochondrial function. *ND4*, nicotinamide adenine dinucleotide dehydrogenase subunit 4; *COX1*, cytochrome c oxidase 1; ATP, adenosine triphosphate; LDH, lactate dehydrogenase; CS, citrate synthase; SDH, succinate dehydrogenase; *SDHA*, succinate dehydrogenase flavoprotein subunit A; *IR*, insulin receptor; *PI3K*, phosphatidylinositol 3-kinase; *AKT*, serine/threonine kinase 1; *FoxO1*, forkhead box protein O1; Lbc, the light brown control group; Lbr, the light brown with resveratrol treatment group. Asterisk (*) indicates a significant difference (*P* < 0.05)

Figure 8 shows the effects of resveratrol supplementation on uterine mitochondrial function. Resveratrol supplementation elevated the expression levels of mtDNA *ND4* and *COX1* (*P* < 0.05). Compared to the Lbc group, the Lbr group exhibited lower LDH activity and higher CS activity (*P* < 0.05). Additionally, the Lbr group upregulated the expression levels of *CS* and *SDHA* genes, while downregulating those of *IR* and *PI3K* (*P* < 0.05). However, the supplementation with resveratrol did not significantly affect the ATP level, SDH activity, or the expression levels of *AKT* and *FoxO1* genes (*P* > 0.05).

### Relative mRNA and protein expression related to the SIRT1/PGC-1α/ALAS1 pathway

**Fig. 9:**
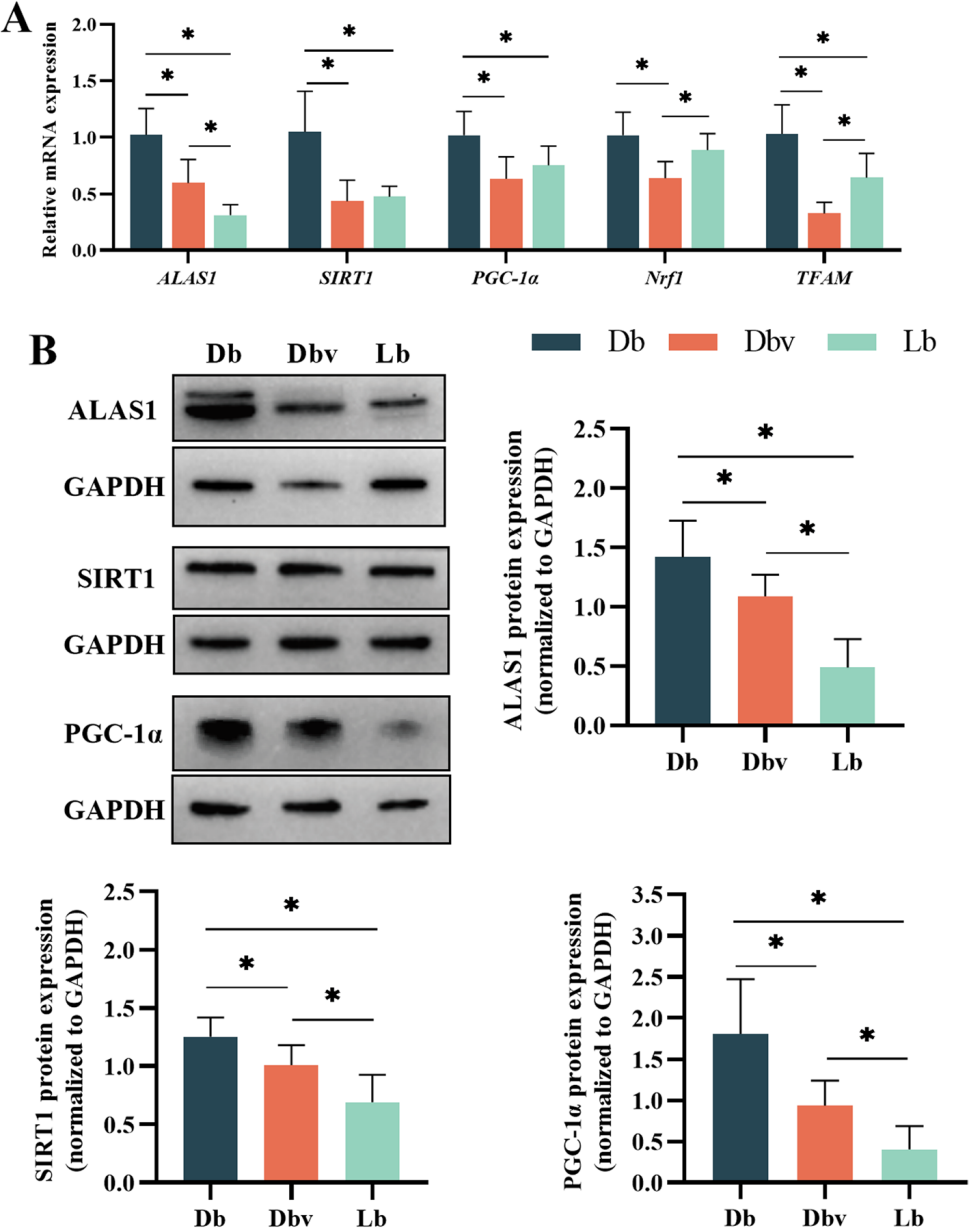
Effects of vanadium addition on the expression of SIRT1/PGC-1α/ALAS1 pathway (*n* = 8). **A** The relative mRNA expression. **B** The relative protein expression. ALAS1, δ-aminolevulinic acid synthase1; SIRT1, silencing information regulator 1; PGC-1α, peroxisome proliferator-activated receptor γ coactivator 1α; *Nrf1*, nuclear factor E2-related factor 1; *TFAM*, mitochondrial transcription factor A; GAPDH, glyceraldehyde-3-phosphate dehydrogenase; Db, the dark brown group; Dbv, the vanadium-treated group; Lb, the light brown group. Asterisk (*) indicates a significant difference (*P* < 0.05)

Effects of vanadium addition on the expression of SIRT1/PGC-1α/ALAS1 pathway are plotted in Fig. 9. Compared to the Db group, the expression levels of *ALAS1*, *SIRT1*, *PGC-1α*, and *TFAM* genes were reduced in both Dbv and Lb groups, while *Nrf1* expression was decreased only in the Dbv group (*P* < 0.05). Additionally, the Lb group exhibited a lower expression level of *ALAS1* gene but higher expression levels of *Nrf1* and *TFAM* genes compared to the Dbv group (*P* < 0.05). Similarly, the protein expression levels of ALAS1, SIRT1, and PGC-1α were significantly reduced in the Dbv group compared to the Db group, yet remained higher than those in the Lb group (*P* < 0.05).

**Fig. 10:**
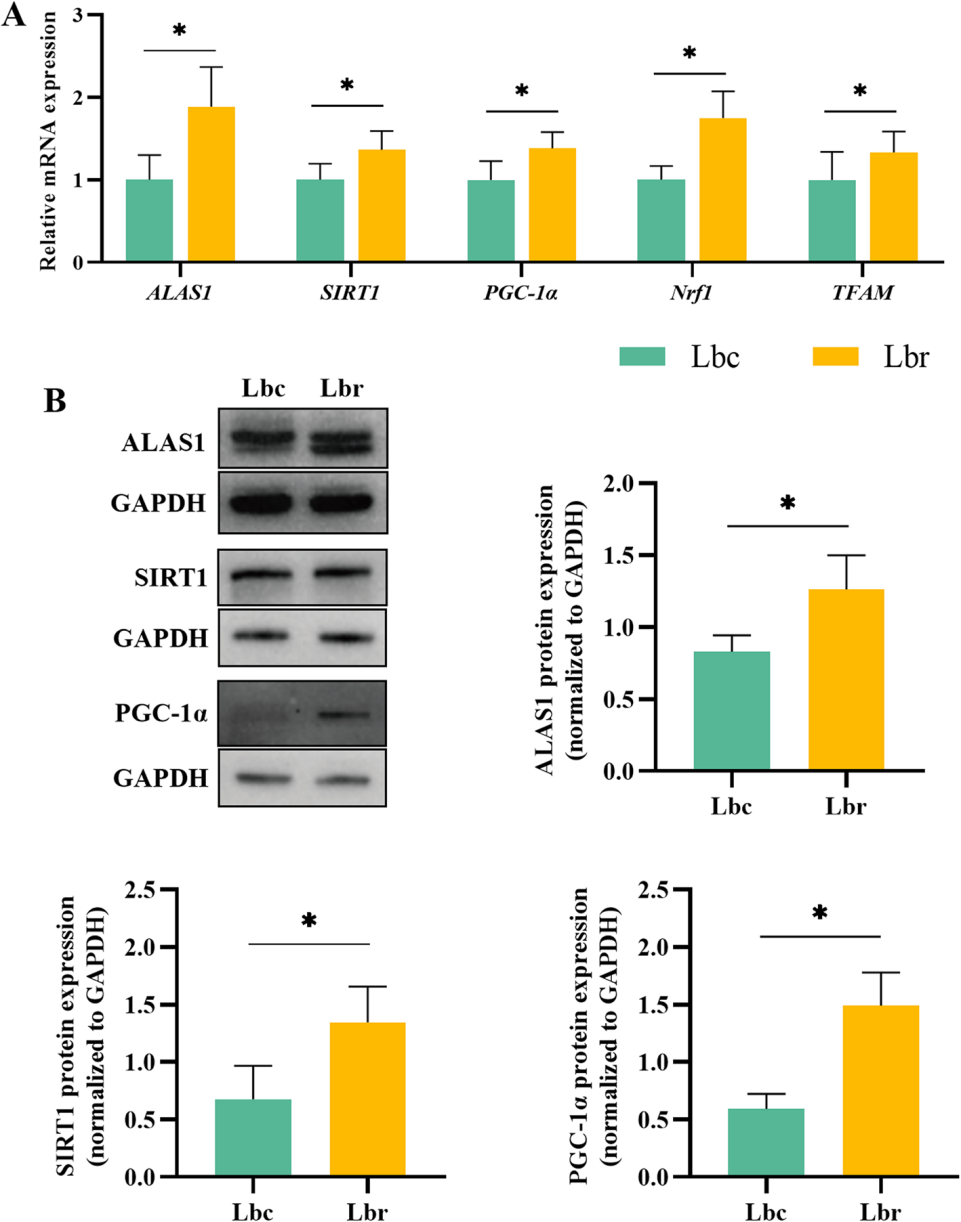
Effects of dietary resveratrol supplementation on the expression of SIRT1/PGC-1α/ALAS1 pathway (*n* = 8). **A** The relative mRNA expression. **B** The relative protein expression. ALAS1, δ-aminolevulinic acid synthase1; SIRT1, silencing information regulator 1; PGC-1α, peroxisome proliferator-activated receptor γ coactivator 1α; *Nrf1*, nuclear factor E2-related factor 1; *TFAM*, mitochondrial transcription factor A; GAPDH, glyceraldehyde-3-phosphate dehydrogenase; Lbc, the light brown control group; Lbr, the light brown with resveratrol treatment group. Asterisk (*) indicates a significant difference (*P* < 0.05)

The expression levels of *Nrf1* and *TFAM* genes were significantly elevated in the Lbr group compared to the Lbc group (Fig. 10A, *P* < 0.05). In addition, both gene and protein expression levels of ALAS1, SIRT1, and PGC-1α were significantly upregulated in the Lbr group relative to the Lbc group (Fig. 10, *P* < 0.05).

## Discussion

This study investigated the impact of redox status on eggshell color in laying hens. As a central organ for metabolism and detoxification, the liver plays a crucial role in systemic redox regulation [30]. In this study, vanadium exposure significantly reduced the activities of major antioxidant enzymes, including GSH-Px, CAT, and T-SOD, in the liver, indicating impaired antioxidant defense capacity, which is consistent with a previous report [31]. This decline in enzymatic activity may be attributed to the downregulation of *CAT* and *MnSOD* gene expression, which weakens the ability to scavenge reactive oxygen species (ROS) and leads to cumulative oxidative damage [32]. Vanadium-induced oxidative stress activated the PI3K/AKT-mediated Nrf2 signaling pathway, potentially contributing to the mitigation of oxidative damage [33]. However, this protective response appeared insufficient, as oxidative stress extended beyond the liver. In the uterus, a key site for pigment biosynthesis and deposition, vanadium exposure also resulted in elevated T-AOC activity, increased *Nrf2*, *HO-1*, and *FECH* expression, further supporting the presence of systemic redox imbalance [32, 34]. Notably, the uterus is responsible for producing PpIX, the primary pigment contributing to brown eggshell coloration. In this study, vanadium treatment reduced PpIX concentrations in both the uterus and the eggshell itself. These findings suggest that oxidative stress in the uterus may impair PpIX biosynthesis and/or deposition, leading to increased L* values and visibly depigmented eggshells. Additionally, even in the absence of vanadium treatment, hens that naturally laid light-brown eggs presented signs of oxidative stress, suggesting that endogenous factors or prolonged environmental challenges may contribute to redox imbalance and serve as potential contributors to eggshell color fading [2, 35]. Consistently, supplementation with antioxidants such as vitamin C can alleviate oxidative stress and partially restore eggshell pigmentation [5]. In addition, yeast β-glucan, which also possesses antioxidant properties, has been shown to significantly improve eggshell color in laying hens [36]. Although vanadium may influence calcium metabolism and potentially affect eggshell quality [37], in this study, the 20-day vanadium treatment did not alter eggshell breaking strength or thickness but significantly affected eggshell color. These findings are consistent with our previous studies showing that vanadium primarily impacts eggshell pigmentation [5, 22]. Longer-term vanadium exposure might affect eggshell quality through other pathways, which warrants future investigation.

The reduction in PpIX concentrations in both the uterus and the eggshell may be partly attributed to enhanced degradation. In the vanadium-treated group, we observed upregulation of *FECH* and *HO-1* in the uterus, indicating that acute oxidative stress triggered the sequential metabolism of PpIX into heme and subsequently into biliverdin, as part of the antioxidant defense mechanism [4, 38]. Given its antioxidant properties, biliverdin was likely consumed during the redox response, resulting in decreased levels in the uterus. A decrease in biliverdin content was also observed in hens laying naturally light-colored eggs, further supporting the hypothesis that oxidative stress contributes to biliverdin depletion.

Since our previous research identified impaired PpIX production as a key factor contributing to reduced eggshell color [8], the present study further explored how redox status affects PpIX biosynthesis. Given that mitochondria serve as the primary site of PpIX synthesis and are susceptible to oxidative stress [9, 39], we further examined mitochondrial function under vanadium-induced oxidative stress by assessing the expression of mtDNA and energy metabolism-related enzymes and genes. In the present study, the reduced mRNA expression levels of *ND4* and *COX1* in the vanadium-treated group indicate impaired functionality of the mitochondrial electron transport chain (ETC) [40]. *ND4*, a critical subunit of Complex I, plays a pivotal role in initiating electron transfer and proton pumping [41]. Its downregulation impedes these initial processes, while the decreased expression of *COX1*, an essential component of Complex IV, compromises the terminal step of the electron transfer to oxygen and the establishment of the mitochondrial membrane potential [42, 43]. These disruptions collectively lead to inefficient oxidative phosphorylation and reduced ATP production, as corroborated by the significantly decreased ATP content observed in the vanadium-treated group. Additionally, the decreased activity of CS, a rate-limiting enzyme in the tricarboxylic acid (TCA) cycle, further underscores the adverse effects of oxidative stress on mitochondrial energy metabolism [44]. The diminished CS activity likely slows the TCA cycle, limiting the production of NADH and FADH_2_, critical electron donors for the ETC, and thereby exacerbating the energy deficit [45]. Although we observed an upregulation in the activity of SDH, which functions as both a TCA cycle enzyme and Complex II of the ETC [46], this response may represent a compensatory mechanism aimed at preserving residual electron flow and ATP production. However, this compensation appears insufficient to counteract the overall mitochondrial dysfunction. These findings indicate that vanadium-induced oxidative stress disrupts mitochondrial energy metabolism at multiple levels, such as affecting the ETC, the TCA cycle, and associated enzymatic activities, thereby leading to ATP depletion and the reduction in PpIX biosynthesis.

As ATP levels declined, the activation of the IR/PI3K signaling pathway was detected, possibly reflecting an adaptive cellular response to increase glucose uptake [47, 48]. Concurrently, increased mRNA expression of key glycolytic enzymes, including *HK* and *PK*, suggests a metabolic shift toward glycolysis as an alternative means of ATP generation [49]. The elevated LDH activity further supports the enhancement of glycolytic flux. While glycolysis provides a rapid, oxygen-independent source of energy under conditions of mitochondrial impairment, its ATP yield is significantly lower than that of oxidative phosphorylation [50]. Moreover, prolonged reliance on glycolysis may lead to lactate accumulation and metabolic imbalance, potentially compromising cellular homeostasis. Thus, insufficient ATP supply under oxidative stress may drive a metabolic shift toward glycolysis, ultimately limiting the mitochondrial energy required for effective PpIX synthesis.

Based on the findings that oxidative stress impairs mitochondrial function and reduces ATP production, we further investigated the potential molecular mechanisms by which these alterations inhibit PpIX biosynthesis. Adequate ATP is not only essential for general mitochondrial function but also directly supports the enzymatic activity of ALAS1, the rate-limiting enzyme in the PpIX biosynthesis pathway [51]. Insufficient ATP availability under oxidative stress likely impairs the energy-dependent transport and conversion of precursors required for ALAS1-mediated reactions, thereby disrupting the early steps of PpIX synthesis [52]. In addition, we observed that vanadium-induced oxidative stress significantly suppressed both mRNA and protein levels of ALAS1, indicating transcriptional repression of this key enzyme. This reduction was accompanied by decreased expression of the upstream transcription factors *Nrf1* and PGC-1α, which are known to positively regulate ALAS1 expression [53]. Furthermore, the expression of SIRT1, a redox-sensitive NAD^+^-dependent deacetylase [54], was also diminished. Since SIRT1 activates PGC-1α through deacetylation, its reduction may have indirectly contributed to PGC-1α suppression and the subsequent downregulation of *Nrf1* [55]. The weakened SIRT1/PGC-1α signaling cascade thus impaired mitochondrial biogenesis and reduced ALAS1 expression. Together, these findings suggest that oxidative stress inhibits PpIX synthesis not only by reducing mitochondrial ATP production required for ALAS1 enzymatic activity, but also by disrupting the SIRT1/PGC-1α signaling cascade that governs ALAS1 transcription, ultimately contributing to lighter eggshell pigmentation. Furthermore, hens that naturally produce lighter-colored eggs also exhibited mitochondrial dysfunction and a similar downregulation of the SIRT1/PGC-1α/ALAS1 cascade as observed in vanadium-treated hens. This similarity suggests that oxidative stress-induced mitochondrial dysfunction and suppression of the ALAS1-associated regulatory cascade may represent a shared molecular pathway that compromises PpIX biosynthesis and leads to reduced eggshell pigmentation.

To further validate the critical role of redox status and the SIRT1/PGC-1α/ALAS1 signaling cascade in regulating eggshell pigmentation, Exp. 2 explored whether pharmacological activation of SIRT1 could reverse mitochondrial dysfunction and improve PpIX biosynthesis. Resveratrol, a natural polyphenol found in grapes, blueberries, and other plants, was selected due to its well-documented antioxidant and SIRT1-activating properties [56]. In this experiment, resveratrol was administered to hens naturally laying light-brown eggs, which, as shown previously, exhibited signs of oxidative stress, mitochondrial impairment, and suppressed PpIX synthesis. Following resveratrol treatment, oxidative stress markers significantly improved in both liver and uterus tissues. In the liver, MDA levels were reduced to near-normal levels, and CAT activity markedly increased. Similarly, in the uterus, MDA accumulation declined, accompanied by a significant increase in the expression and activity of antioxidant enzymes, including CAT, MnSOD, GSH-Px, and T-SOD. These results indicate a systemic restoration of redox balance, likely mediated by Nrf2/HO-1 pathway activation [57].

Importantly, this improved redox status in both tissues coincided with a significant upregulation of uterine SIRT1 expression. As SIRT1 activity increased, PGC-1α deacetylation and activation were enhanced, which in turn upregulated the expression of *Nrf1*, *Nrf2*, and *TFAM*, promoting mitochondrial biogenesis and transcriptional activity [58, 59]. As a result, mitochondrial function was restored, with elevated *ND4* and *COX1* expression indicating improved performance of electron transport chain Complexes I and IV. Concurrently, increased CS activity and *SDHA* expression accelerated the TCA cycle, improving NADH/FADH_2_ supply to the ETC [45]. The reversal of glycolytic reliance, evidenced by decreased IR/PI3K signaling and LDH activity, further supported a metabolic shift back to oxidative phosphorylation as the primary energy production mode [47, 48]. However, total ATP content did not show a significant increase. A possible explanation is that ATP was consumed in parallel by biosynthetic processes, including PpIX synthesis, although this remains to be further confirmed. Notably, enhanced mitochondrial energy metabolism may provide sufficient ATP to support the ALAS1-catalyzed rate-limiting step of the PpIX pathway. At the same time, activation of the SIRT1/PGC-1α axis markedly increased ALAS1 mRNA and protein expression, further enhancing PpIX biosynthesis efficiency. This molecular restoration translated into physiological improvement, as reflected by the significant increase in PpIX content in the uterus and eggshell, along with a reduction in L* values, indicating deeper brown pigmentation. Together, these results underscore the critical role of the SIRT1/PGC-1α/ALAS1 pathway in coupling redox balance to mitochondrial function and PpIX synthesis. Resveratrol’s capacity to restore this pathway under oxidative stress not only reversed pigment loss but also further confirm a shared mechanism behind eggshell color regulation. Additionally, it should be noted that all hens used in this study were aged, which may independently influence mitochondrial function and redox homeostasis. Age-related declines in mitochondrial function and increased oxidative stress could partially contribute to reduced PpIX biosynthesis and eggshell depigmentation, even in the absence of vanadium exposure. Therefore, while our results demonstrate the effects of vanadium-induced oxidative stress and the restoration by resveratrol, the observed changes may be partially influenced by the natural aging process. Future studies including younger hens would help disentangle age-related effects from treatment-specific effects.

## Conclusions

In conclusion, the oxidative stress leads to eggshell depigmentation by impairing mitochondrial function and downregulating the SIRT1/PGC-1α/ALAS1 signaling pathway, resulting in reduced PpIX biosynthesis. Specifically, vanadium-induced or endogenous oxidative stress disrupts mitochondrial energy metabolism and suppresses the expression and activity of key regulatory components in this pathway, while resveratrol alleviates oxidative damage and restores mitochondrial function and ALAS1-driven PpIX synthesis through reactivation of the SIRT1/PGC-1α axis.

## Supplementary Information

Additional file 1: Table S1. Composition of the basal diet. Table S2. Sequences of the primers. Table S3. Details of primary antibodies used in Western blot analysis. Table S4. Effect of vanadium addition on laying performance. Table S5. Effect of dietary resveratrol supplementation on laying performance.

Additional file 2. Original gel and blot images for Fig. 9B and Fig. 10B.

## Data Availability

All data generated or analyzed during this study are available within the published article and its supplementary materials.

## Abbreviations

*AKT*: Serine/threonine kinase 1
ALAS1: δ-Aminolevulinic acid synthase1
ATP: Adenosine triphosphate
CAT: Catalase
*COX1*: Cytochrome c oxidase 1
CS: Citrate synthase
Db: Dark brown
Dbv: Vanadium-treated
*FECH*: Ferrochelatase
*FoxO1*: Forkhead box protein O1
GAPDH: Glyceraldehyde-3-phosphate dehydrogenase
GSH-Px: Glutathione peroxidase
*HK*: Hexokinase
*HO-1*: Heme oxygenase 1
*IR*: Insulin receptor
Lb: Light brown
Lbc: Light brown control
Lbr: Light brown with resveratrol treatment
LDH: Lactate dehydrogenase
*LDHA*: Lactate dehydrogenase A
MDA: Malondiadehyde
*MnSOD*: Manganese superoxide dismutase
*ND4*: Nicotinamide adenine dinucleotide dehydrogenase subunit 4
*Nrf1*: Nuclear factor E2-related factor 1
*Nrf2*: Nuclear factor E2-related factor 2
PGC-1α: Peroxisome proliferator-activated receptor γ coactivator 1α
*PI3K*: Phosphatidylinositol 3-kinase
*PK*: Pyruvate kinase
PpIX: Protoporphyrin IX
SDH: Succinate dehydrogenase
*SDHA*: Succinate dehydrogenase flavoprotein subunit A
SIRT1: Silencing information regulator 1
T-AOC: Total antioxidant capacity
*TFAM*: Mitochondrial transcription factor A
T-SOD: Total superoxide dismutase
