# Supplementary material for: Redox status regulates eggshell color by modulating protoporphyrin IX biosynthesis via the SIRT1/PGC-1α/ALAS1 axis in brown-shelled hens
Source: J Anim Sci Biotechnol. 2025 Nov 21;16:157. doi: 10.1186/s40104-025-01292-9 (PMC12636190; doi:10.1186/s40104-025-01292-9)
Supplement: Supplementary file 1 — Additional file 1: Table S1. Composition of the basal diet. Table S2. Sequences of the primers. Table S3. Details of primary antibodies used in Western blot analysis. Table S4. Effect of vanadium addition on laying performance. Table S5. Effect of dietary resveratrol supplementation on laying performance. [file 40104_2025_1292_MOESM1_ESM.docx]

**Table S1** Composition of the basal diet

| Ingredients, % |  | Nutrient levels, % |  |
| --- | --- | --- | --- |
| Corn | 65.50 | Metabolizable energy, MJ/kg | 11.16 |
| Soybean meal | 23.50 | Crude protein | 16.50 |
| Limestone | 8.93 | Total phosphorus | 0.54 |
| Sodium chloride | 0.30 | Available phosphorus | 0.33 |
| Dicalcium phosphate | 1.35 | Calcium | 3.31 |
| 50% Choline chloride | 0.10 | Lysine | 0.86 |
| DL-Methionine | 0.10 | Methionine | 0.37 |
| Premix^1^ | 0.22 | Cystine | 0.26 |

^1^ Premix provided the following per kg of the diet: Cu, 8 mg; Zn, 66 mg; Fe, 60 mg; Mn, 65 mg; Se, 0.3 mg; I, 1 mg; Vitamin A, 9,500 IU; vitamin D_3_, 4,125 IU; vitamin E, 15 IU; vitamin K, 2 mg; thiamine, 1 mg; riboflavin, 8.5 mg; pyridoxine, 8 mg; vitamin B_12_, 0.02 mg; pantothenic acid, 50 mg; niacin, 32.5 mg; biotin, 2 mg; folic acid, 5 mg.

**Table S2** Sequences of the primers

| Genes | Primer Sequence (5′-3′) | Accession No. |
| --- | --- | --- |
| *β-actin* | F:ATGATATTGCTGCGCTCGTT | L08165 |
|  | R:TCTTTCTGGCCCATACCAACC |  |
| *ND4* | F:CGCAGGCTCCATACTACTCG | NC_040970.1 |
|  | R:TTAGGGCACCTCATAGGGCT |  |
| *COX1* | F:CCATACTACTTACCGACCGCAACC | NC_040970.1 |
|  | R:GTGTCTACGTCCATTCCGACTGTG |  |
| *ALAS1* | F:GGTGGACAGGAAAGGTAAAGA | NM_001018012 |
|  | R:ACTGGTCATACTGGAAGGTG |  |
| *NRF1* | F:ACAGCAACAGACCACAACCACAC | NM_001030646 |
|  | R:CTGCTTCTGCTAACGATGCTACCG |  |
| *NRF2* | F:GAGCAAAGAGCAAAAGCTCTG | NM_001396905 |
|  | R: GGCAGCCACTTTATTCTTGC |  |
| *HO-1* | F:TCCATCTCAAGGGCATTCA | NM_205344 |
|  | R:TTGGCAAGAAGCATCCAGA |  |
| *FOXO1* | F:TCTGGTCAGGAGGGAAATGG | NM_204328 |
|  | R:GCTTGCAGGCCACTTTGAG |  |
| *PI3K* | F:ACCAAAACAAGGGAGTTTGAAAGG | NM_001004410 |
|  | R:TCCAGTCCATTTTTGTTGTCCAGG |  |
| *IR* | F:CGGAACTGCATGGTTGCA | XM_001233398 |
|  | R:TCTCTGGTCATGCCGAAGTCT |  |
| *SIRT1* | F:AAGACCTGCTCCCAGAAACG | XM_046920052 |
|  | R:ACAGCAAGGCGTGCATAGAT |  |
| *HK* | F:TTCGACCACATCGTCCACTG | NM_001396482 |
|  | R:ACCACGTCCAGGTCAAACTC |  |
| *PK* | F:AGCAGCAGGAGACACCGAAC | NM_205469 |
|  | R:TGAGGCGGGCAACATTCAT |  |
| *CS* | F:TACTACACGGTGCTCTTCGG | XM_040693722 |
|  | R:CGGATCCTGCCGGATTTGTAG |  |
| *SDHA* | F:TCTGTCCATGGTGCTAATCG | NM_001277398 |
|  | R:TGGTTTAATGGAGGGGACTG |  |
| *CAT* | F:GCATGTCCGTTTCAGGAGAT | NM_001031215 |
|  | R:CGCCATAGTCAGGATGAACA |  |
| *MNSOD* | F:CACTCTTCCTGACCTGCCTTAC | NM_204211 |
|  | R:TAGACGTCCCTGCTCCTTATTA |  |
| *PGC-lα* | F:CGCTTCACTGTTATGAGTCC | NM_001006457 |
|  | R:TCTGTCTCTCCCTTTGTTTGG |  |
| *FECH* | F:ACACCACGAATTCAGGAGCA | NM_204196 |
|  | R:AGCAGTTTCACCATGCCTTCT |  |
| *AKT* | F:AGGAGGAAGAGATGATGGAT | NM_205055 |
|  | R:GAATGGATGCCGTGAGTT |  |
| *TFAM* | F:CGAGGAAGCAAGGAAGACAGACTG | NM_204100 |
|  | R:CCTGAGCAAGCTGAAGGTATGGC |  |

^1^ F, forward; R, reverse; *ALAS1*, δ-aminolevulinic acid synthase 1; *ND4*, NADH dehydrogenase subunit 4; *COX1*, cytochrome c oxidase subunit 1; *GAPDH*, glyceraldehyde-3-phosphate dehydrogenase; *NRF1*, nuclear factor E2-related factor 1; *NRF2*, nuclear factor E2-related factor 2; *HO-1*, heme oxygenase-1; *FOXO1*, forkhead box protein O1; *PI3K*, phosphatidylinositol-4,5-bisphosphate 3-kinase; *IR*, insulin receptor; *SIRT1*, sirtuin 1; *HK*, hexokinase; *PK*, pyruvate kinase; *CS*, citrate synthase; *SDHA*, succinate dehydrogenase flavoprotein subunit A; *CAT*, catalase; *MNSOD*, manganese superoxide dismutase; *PGC-1α*, peroxisome proliferator-activated receptor gamma coactivator 1-alpha; *FECH*, ferrochelatase; *AKT*, protein kinase B; *TFAM*, mitochondrial transcription factor A.

^2^ *COX1* and *ND4* were used to amplify fragment of mitochondrial DNA.

**Table S3** Details of primary antibodies used in Western blot analysis

| Antibody | Manufacturer | Catalog number | Host species | Working dilution | Sequence homology |
| --- | --- | --- | --- | --- | --- |
| SIRT1 | abcam | ab189494 | Rabbit | 1/1000 | 96.7% |
| PGC-1α | abcam | ab54481 | Rabbit | 1/1000 | 95.2% |
| ALAS1 | abcam | ab154860 | Rabbit | 1/5000 | 86.7% |
| GAPDH | abcam | ab181602 | Rabbit | 1/10000 | 94.0% |

^1^ ALAS1, δ-aminolevulinic acid synthase1; GAPDH, glyceraldehyde-3-phosphate dehydrogenase; PGC-1α, peroxisome proliferator-activated receptor γ coactivator 1α; SIRT1, silencing information regulator 1.

**Table S4** Effect of vanadium addition on laying performance

| Items | Dark brown | Dark brown + vanadium | Light brown |
| --- | --- | --- | --- |
| Average daily feed intake, g | 105.5 | 94.50 | 98.50 |
| Laying rate, % | 95.31 | 95.00 | 94.38 |

**Table S5** Effect of dietary resveratrol supplementation on laying performance

| Items | Light brown | Light brown + resveratrol |
| --- | --- | --- |
| Average daily feed intake, g | 110.83 | 102.50 |
| Laying rate, % | 92.26 | 93.13 |
