# Supplementary figures and images for: Redox status regulates eggshell color by modulating protoporphyrin IX biosynthesis via the SIRT1/PGC-1α/ALAS1 axis in brown-shelled hens
Source: J Anim Sci Biotechnol. 2025 Nov 21;16:157. doi: 10.1186/s40104-025-01292-9 (PMC12636190; doi:10.1186/s40104-025-01292-9)

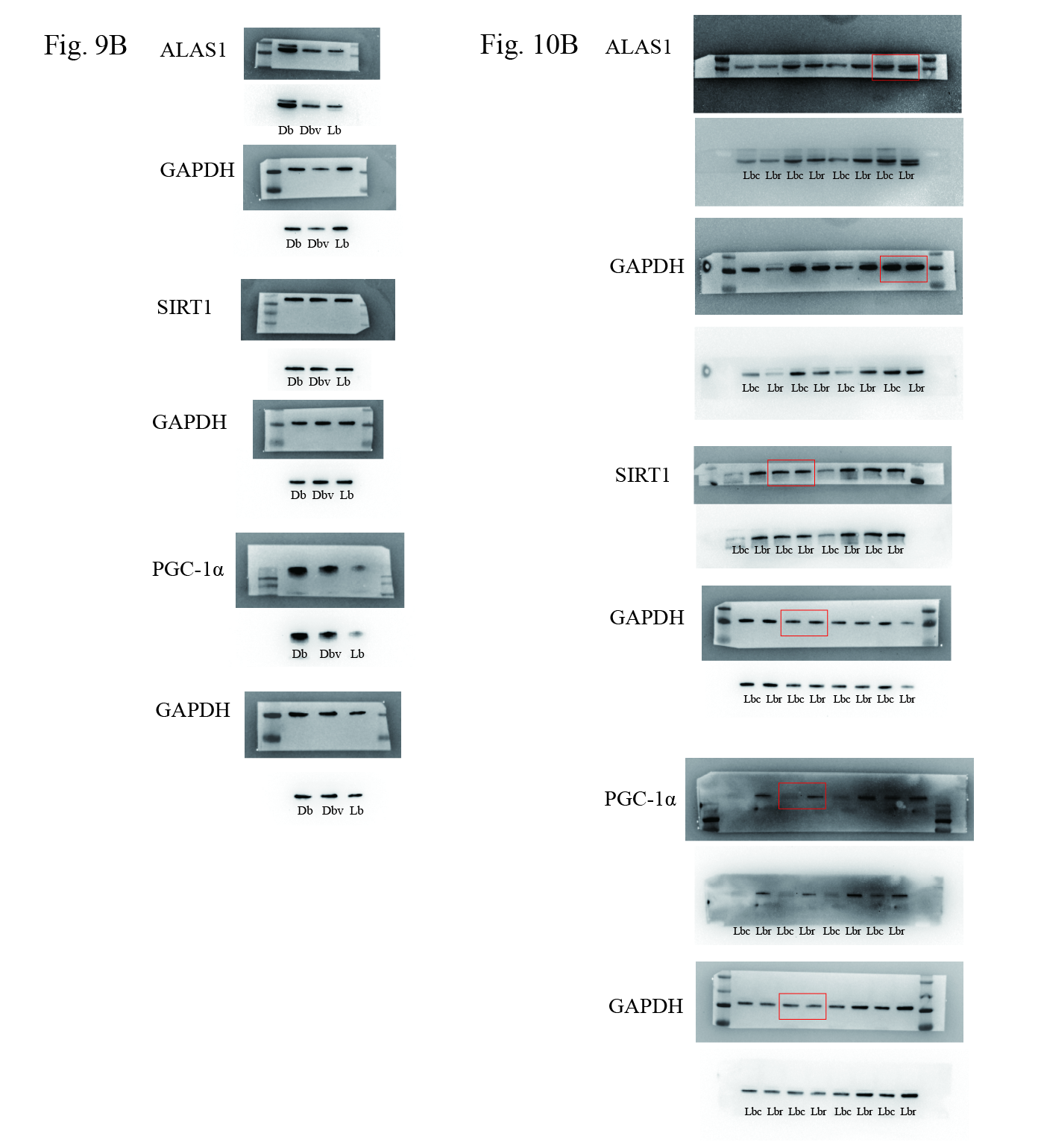

Supplement: Supplementary file 2 — Additional file 2. Original gel and blot images for Fig. 9B and Fig. 10B. [file 40104_2025_1292_MOESM2_ESM.tif]
